# Temporal and masseter muscle evaluation by MRI provides information on muscle mass and quality in acromegaly patients

**DOI:** 10.1007/s11102-024-01422-y

**Published:** 2024-07-05

**Authors:** Federico Gatto, Angelo Milioto, Giuliana Corica, Federica Nista, Claudia Campana, Anna Arecco, Lorenzo Mattioli, Lorenzo Belluscio, Bianca Bignotti, Diego Ferone, Alberto Stefano Tagliafico

**Affiliations:** 1 Endocrinology Unit, IRCCS Ospedale Policlinico San Martino, Largo Rosanna Benzi, 10, Genoa, 16132 Italy; 2 Endocrinology Unit, Department of Internal Medicine and Medical Specialties (DIMI), Centre of Excellence for Biomedical Research (CEBR), University of Genova, Genoa, Italy; 3 Radiology Section, Department of Health Sciences (DISSAL), University of Genova, Genoa, Italy; 4 Department of Radiology, IRCCS Ospedale Policlinico San Martino, Genoa, Italy

**Keywords:** Acromegaly, Skeletal muscle, Temporal, Masseter, Insulin-like growth factor-1, Magnetic resonance imaging

## Abstract

**Purpose:**

The impact of GH/IGF-1 levels on skeletal muscle in acromegaly is still controversial. Temporal (TMT) and masseter muscle (MMT) thickness has been recently demonstrated as a reliable measure of muscle mass. We aimed to investigate the relationship between TMT, MMT and clinical/biochemical characteristics in patients with acromegaly.

**Methods:**

Single center retrospective longitudinal study including 69 patients with at least one available brain/sella turcica MRI and matched clinical data. TMT, MMT, and muscle fatty infiltration (modified Goutallier score) were evaluated in all patients at baseline (first available MRI) and over time (182 MRIs analyzed).

**Results:**

At baseline, both TMT and MMT were higher in males than females (*p* = 0.001 and *p* = 0.016, respectively). TMT and MMT were positively associated (β 0.508, *p* < 0.001), and they were positively correlated with IGF-1 xULN (TMT, *p* = 0.047; MMT, *p* = 0.001). MMT had a positive correlation with patients’ weight (*p* = 0.015) and height (*p* = 0.006). No correlation was found between TMT, MMT and the presence of hypogonadism. Considering all available MRIs, sex and IGF-1 xULN were significant determinants of TMT and MMT at multivariable analysis (female sex: β -0.345/-0.426, *p* < 0.001; IGF-1 xULN: β 0.257/0.328, *p* < 0.001). At longitudinal evaluation, uncontrolled patients at baseline showed a significant reduction of MMT over time (*p* = 0.044). Remarkable fatty infiltration was observed in 34–37% of MRIs; age was the main determinant (temporal muscle: OR 1.665; *p* = 0.013; masseter muscle: OR 1.793; *p* = 0.009).

**Conclusion:**

Male patients with higher IGF-1 values have thicker temporal and masseter muscles, suggesting that sex and IGF-1 have a significant impact on muscle mass in acromegaly.

**Supplementary Information:**

The online version contains supplementary material available at 10.1007/s11102-024-01422-y.

## Introduction

Acromegaly is a rare and severe endocrine disorder due to the prolonged exposure to high circulating levels of GH and IGF-1, caused in more than 95% of cases by a GH-secreting pituitary adenoma [1, 2].

As concerns skeletal muscle, a shift of amino-acid metabolism towards protein synthesis and decreased protein breakdown has been described in patients with acromegaly [3, 4]. However, the eventual presence of higher skeletal muscle mass does not necessarily translate into increased strength and function [5, 6], since acromegaly is characterized by myopathy with weakness, pain and reduced muscular endurance [7, 8]. Furthermore, an increase in intramuscular fat content, due to increased lipolysis in extra-muscular deposits and increased muscle uptake of lipids during the active phase of the disease, has been described [9, 10].

Whole-body magnetic resonance imaging (WB-MRI), dual-energy X-ray absorptiometry (DXA), computed tomography (CT) and bioelectrical impedance analysis (BIA) techniques have been used to evaluate muscle mass in patients with acromegaly, with varying results [11–15].

Recent studies have shown that the morphometric analysis of the temporalis muscle, performed measuring temporal muscle thickness (TMT) using both CT and MRI techniques, strongly correlates with the measure of psoas muscle, as well as with the patients’ functional status/prognosis, thus representing a reliable measure of skeletal muscle mass in various clinical contexts (e.g. melanoma brain metastases, glioblastoma) [16–19].

In this study we aimed to investigate whether in patients with acromegaly the measure of two craniofacial muscles (temporal and masseter muscle thickness) correlates with general and clinical characteristics known to be associated with skeletal muscle mass (such as sex and age), as well as with hormonal values (e.g. age-adjusted IGF-1).

## Patients and methods

### Study design and patients

Single center retrospective longitudinal study carried out at the Endocrinology Unit of the IRCCS Ospedale Policlinico San Martino (Genoa, Italy). The study was conducted in accordance with the recommendations of the Declaration of Helsinki and all patients gave written consent to use clinical data for research purposes. The study received approval from the local Ethical Committee (IRB number 245/2023).

Sixty-nine patients with an established diagnosis of acromegaly were included. GH and IGF-1 assays used in this study have been recently described in detail [20]. In line with recent consensus statements, we considered as reaching biochemical control those subjects with age-adjusted IGF-1 values ≤ 1 the upper limit of normality (ULN), defined as controlled patients [21, 22].

Inclusion criteria were: (i) availability of at least of one MRI of the brain/sella turcica, in which the measurement of temporal and/or masseter muscle thickness was feasible; (ii) demographics and general patient characteristics, information about time from diagnosis, and treatment path at the time of first available MRI. No additional exclusion criteria were applied.

A total of 182 brain/sella turcica MRIs were collected. Forty-eight out of 69 patients had more than one MRI available for evaluation. In detail, 1 MRI: 69 patients, 2 MRIs: 48/69 (70%), 3 MRIs: 32/69 (46%), 4 MRIs: 16/69 (23%), 5 MRIs: 9/69 (13%), 6 MRIs: 6/69 (9%), 7 MRIs: 2/69 (3%).

For each available MRI, data collected on temporal and masseter muscle thickness were correlated with disease-specific hormonal values (GH, absolute IGF-1, IGF-1 xULN), as well as with the presence of hypogonadism. This latter was defined as the presence of low total testosterone levels and associated symptoms in men, or low levels of estradiol accompanied by the absence of menstrual cycles in women. Women with menopause were included in the hypogonadal group [23].

A maximum interval of one month between MRI and hormonal evaluations (before/after) was considered acceptable for the study purpose.

Additional parameters were collected for each patient at the time of first available MRI (see Table 1).

**Table 1:** Demographics, clinical and biochemical characteristics of the patients included in the study, together with skeletal muscle evaluation at the time of first evaluated MRI.

Data	Number (%)
Patients	69
Sex	F, 44 (64)
Age (median, IQR)	54, 45–64
Body weight (Kg; median, IQR)	80, 68.3–90
Height (m; median, IQR)	1.70, 1.63–1.77
BMI (Kg/m^2^; median, IQR)	27.3, 23.9–30.6
Time from diagnosis to first MRI (months; median, IQR)	21.5, 1-100.8
**Biochemical values**	
GH (µg/L; median, IQR)	2.9, 1.1-9.0
IGF-1 (µg/L; median, IQR)	264, 171–453
IGF-1 (xULN; median, IQR)	1.1, 0.8–1.9
Disease control (IGF-1 ≤ 1 xULN)	31 (44.9)
**Hyperprolactinemia** (yes, %) - CAB treatment	15 (21.7) 5/15
PRL (µg/L; median, IQR)	9.0, 5.8–17.3
**Hypogonadism** (yes, %) - Males/females - TR therapy	41 (59.4) 14/27 2/14
**Hypocortisolism** (yes, %) - HR therapy	7 (10.1) 7/7
**Hypothyroidism** (yes, %) - HR therapy	10 (14.5) 10/10
**AVP deficiency** (yes, %)	0 (0)
**Glucose metabolism**	
Diabetes mellitus (yes, %) - AD therapy	14 (20.3) 10/14
HbA1c (%; median, IQR)	5.8, 5.5–6.4
**Concomitant malignancies** ^**a**^	
Remission	9 (13.0)
Active disease	4 (5.8)
Overall (remission or active disease)	13 (18.8)
**Sleep apnea syndrome** (yes, %)	7 (10.1)
**Treatment modalities**	
Neurosurgery (n, %)	33 (47.8)
Radiotherapy (n, %)	1 (1.4)
Medical therapies (n, %):	34 (49.3)
- fg-SRLs	24 (70.6)
- PAS	2 (5.9)
- PEGV	1 (2.9)
- fg-SRL + PEGV	2 (5.9)
- fg-SRL + CAB	5 (14.7)
**Skeletal muscle evaluation**	
Temporal muscle thickness (mm; median, IQR)	6.1, 5–7
Masseter muscle thickness (mm; median, IQR)	15.1, 12.9–17.7

**Abbreviations.** F, female; IQR, interquartile range; BMI, body mass index; MRI, magnetic resonance imaging; ULN, upper limit of normality; CAB, cabergoline; PRL, prolactin; TR, testosterone replacement; HR, hormone replacement; AVP, arginine vasopressin; AD, antidiabetic; HbA1c, glycated hemoglobin; fg-SRLs, first-generation somatostatin receptor ligands; PAS, pasireotide; PEGV, pegvisomant; mm, millimeter. ^***a***^Presence of cancers/malignant tumors in patients’ clinical history, considered as in remission or still active at the time of first available MRI

Due to the retrospective study design, not all information was available for all patients.

### Analysis of temporal (TMT) and masseter muscle thickness (MMT) and quality

**Fig. 1:**
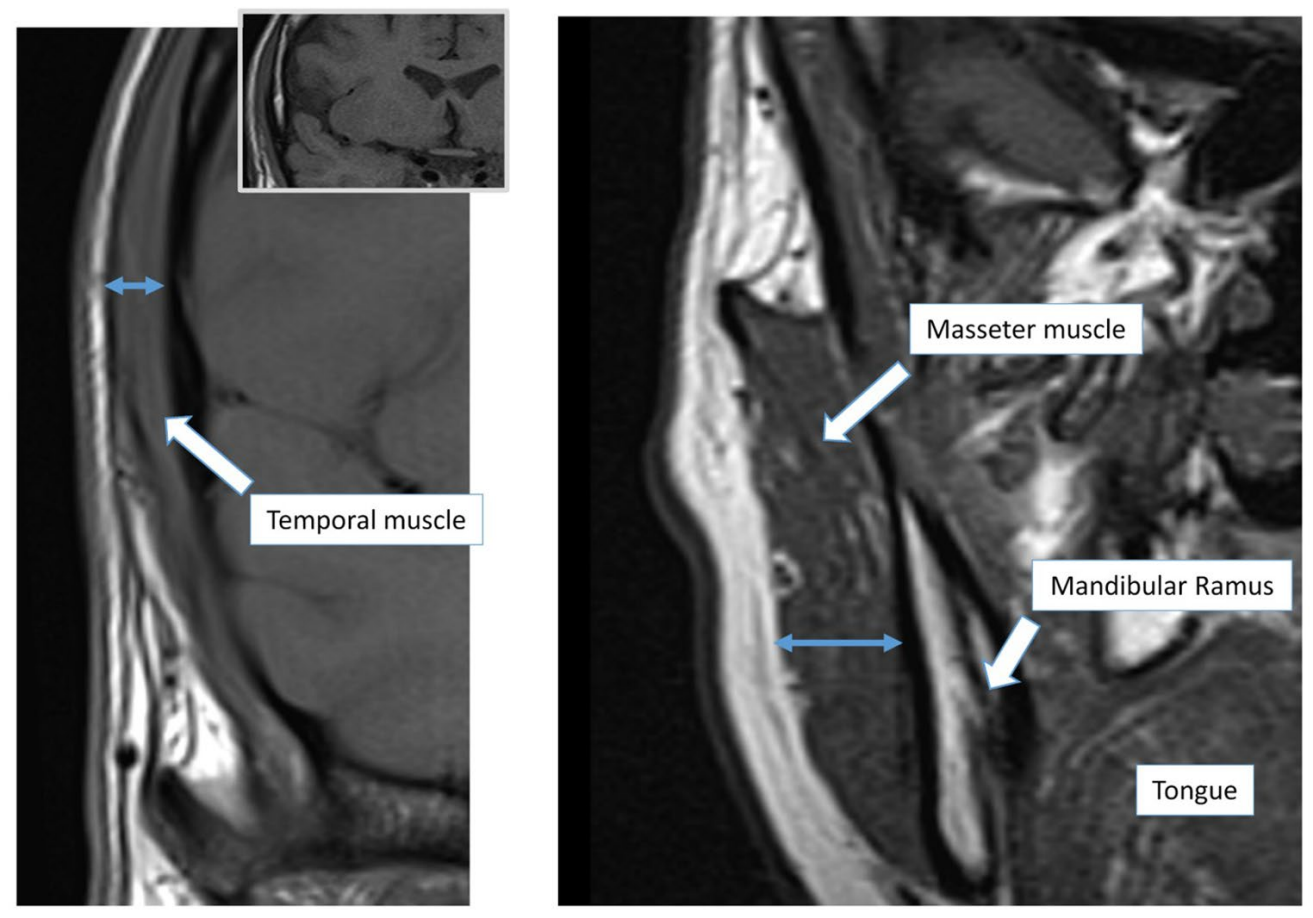
Examples of temporal (panel **A**) and masseter (panel **B**) muscle thickness measurements in two different patients on coronal T1-weighted MRI sequences (double headed arrow). *Abbreviations.* MRI, magnetic resonance imaging

TMT and MMT were calculated on brain/sella turcica MRIs available on patients’ clinical records. Muscular thickness was calculated with electronic calipers on axial and coronal planes of isovoxel (1 × 1 × 1 mm) T1-weighted images as follows: (i) at the level of the orbital roof, perpendicular to the long axis at the point of maximal depth for the temporal muscle [18], and (ii) at the middle of mandibular ramus, perpendicular to the muscle belly for the masseter muscle (Fig. 1). TMT and MMT of both the left and the right side were determined separately, and the mean value was considered for analysis.

Based on the described methods, a proper measurement of TMT and MMT was possible in most but not all MRIs (TMT: 180/182 MRIs [99%]; MMT: 158/182 MRIs [87%]).

Muscles were evaluated for fatty infiltration using a modified Goutallier classification [24–26] as semi-quantitative score: grade 0, normal appearance and early moth-eaten appearance, with scattered small areas of fat comprising < 30% of the volume of the muscles; grade 1, late moth-eaten appearance, with numerous discrete areas of fat with beginning confluence, comprising 30–60% of the volume of the muscles, and washed-out appearance, fuzzy appearance due to confluent areas of fat with muscle still present at the periphery. All measurements on MRIs were done by one musculoskeletal radiologists with more than 10 years of experience in MR imaging (A.S.T.) in consensus with a second musculoskeletal radiologists with 7 years of experience, blinded to clinical features (B.B.).

### Statistical analysis

Statistical analysis was conducted using the SPSS 27.0 software (SPSS, Chicago, IL, USA) for Windows. Continuous variables are expressed as median and interquartile range (IQR), whereas dichotomous variables are reported as frequency and percentage. Comparisons between continuous variables were performed using Mann-Whitney U test and Kruskal-Wallis test. Correlation coefficients were calculated using the Spearman rank order R. Chi square and K tests were used for the comparison between dichotomous variables. To identify the potential predictors of temporal and masseter muscle thickness, as well as of muscle quality, univariable and multivariable logistic regression and linear regression analyses were performed, when appropriate. Significance was accepted for a p-value < 0.05.

## Results

### Patient characteristics at the time of first available MRI

Sixty-nine patients were included in the study, 44 females (64%) and 25 males (36%), with a median age of 54 years (IQR 45–64) at the time of first available MRI. In 19 patients, the first available MRI was performed at the time of diagnosis, while in 50 patients it referred to a follow-up investigation (median time from diagnosis to first analyzed MRI: 21.5 months [IQR 1.0-100.8]).

Median age-adjusted IGF-1 value was 1.1 xULN (IQR 0.8–1.9), and 31 patients (44.9%) were considered as having biochemical control.

Median GH level was 2.9 µg/L (IQR 1.1-9.0); as expected, GH values showed a strong and positive correlation with both absolute IGF-1 (rho: 0.722, *p* < 0.001) and IGF-1 xULN values (rho: 0.681, *p* < 0.001). Noteworthy, total testosterone levels (available in 20 male patients) were inversely correlated with both absolute IGF-1 and IGF-1 xULN values (rho: -0.794 and rho: -0.787, respectively; *p* < 0.001). Controlled patients had significantly higher total testosterone levels (median 330 ng/dl, IQR 251–456) compared to patients with active disease (169 ng/dl, IQR 101–255; *p* = 0.002).

Fifteen patients had hyperprolactinemia (21.7%), which was mild in most cases, thus requiring treatment with the dopamine-agonist cabergoline (CAB) only in five individuals.

Patients’ characteristics are summarized in Table 1 (see Online Resource 1 for more details).

### Temporal and masseter muscle thickness at first available MRI

**Fig. 2:**
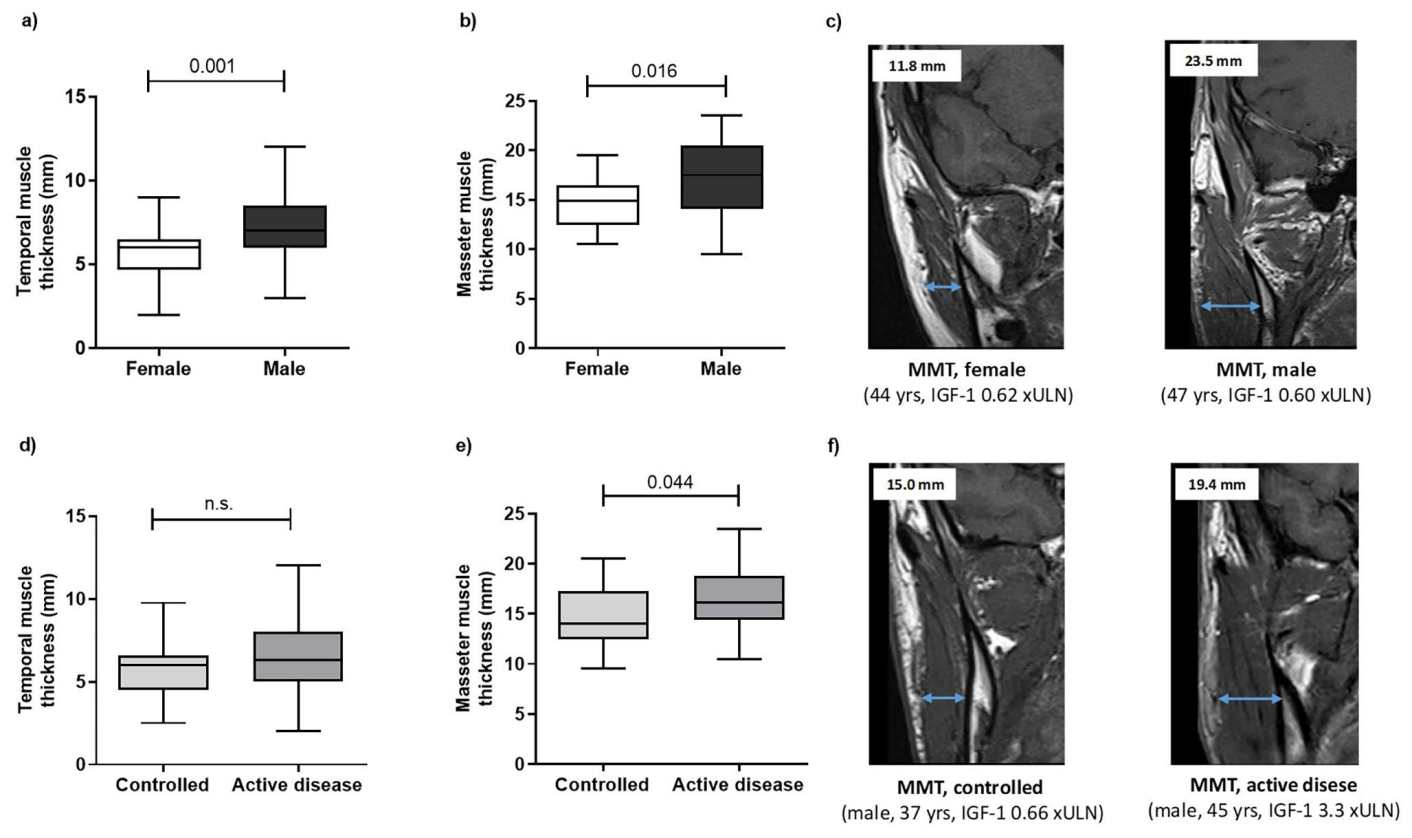
Differences in TMT and MMT measurements according to sex and disease activity in patients with acromegaly (first available MRI). Sex-related differences are depicted in panels **A** and **B**. In panel **C**, two representative images of MMT evaluation in a female (left) and a male (right) patient, matched for age and IGF-1 xULN values, are reported. Differences based on patients’ disease status (controlled [IGF-1 ≤ 1 xULN] vs. active disease [IGF-1 > 1 xULN]) are depicted in panels **D** and **E**. In panel **F**, two representative images of MMT evaluation in two male patients, with different disease activity at the time of radiological examinations, are reported. *Abbreviations.* TMT, temporal muscle thickness; MMT, masseter muscle thickness; MRI, magnetic resonance imaging; yrs, years; ULN, upper limit of normality

Median temporal muscle thickness (TMT) was 6.1 mm (IQR 5–7), being significantly higher in males (6.9 mm, IQR 5.7–7.5) compared to females (6.0 mm, IQR 4.5–6.5; *p* = 0.001) (Fig. 2A). Median masseter muscle thickness (MMT) was 15.1 mm (IQR 12.9–17.7), with significantly higher values in males (17.6 mm, IQR 13.7–20.5) than in females (14.9 mm, IQR 12.5–16.5; *p* = 0.016) (Fig. 2B-C).

Overall, TMT showed a significant positive correlation with MMT (rho: 0.497, *p* < 0.001; *n* = 60). This correlation was maintained when analyzing the subgroup of female patients (rho: 0.529, *p* < 0.001; *n* = 40), while statistical significance was lost in the smaller subgroup of male patients (rho: 0.278, *p* = 0.234; *n* = 20). At univariable regression analysis, TMT and MMT were positively associated (adjusted R^2^ 0.245, B 0.935, β 0.508; *p* < 0.001).

Age of patients at the time of first available MRI did not correlate with either TMT (rho: 0.066, *p* = 0.595) or MMT (rho: -0.046, *p* = 0.720).

Although patients with cancers can present with reduced muscle mass, TMT and MMT were not significantly different in patients with a clinical history of cancer compared to those individuals with no cancer reported in clinical charts (*p* = 0.491 and *p* = 0.767; respectively).

Sleep apnea syndrome (SAS) is frequently encountered in acromegaly, and it was reported in seven patients of our cohort (10%). Taking into account this low number of individuals with SAS, median TMT (*p* = 0.420) and MMT (*p* = 0.068) values did not significantly differ between patients with and without SAS. However, MMT values were numerically higher in patients with SAS compared to those without (18.3 [14.8–20.6] mm vs. 15.0 [12.6–17.5] mm).

### TMT and patients’ characteristics at first MRI

At first available MRI, TMT showed a significant positive correlation with IGF-1 xULN values (rho: 0.249, *p* = 0.047), although TMT values did not significantly differ between active disease and controlled patients (median 6.15 mm, IQR 4.95–7.4 vs. 6.0 mm, IQR 4.5–6.63; *p* = 0.088) (Fig. 2D). This finding was also confirmed setting the IGF-1 cut-off at 1.3 xULN, 2.0 xULN, and even when stratifying patients as of every ULN interval (e.g. 2-fold, 3-fold, etc.). No significant correlations were found between TMT and absolute IGF-1 (rho: 0.227, *p* = 0.070), GH (rho: 0.216, *p* = 0.124), or total testosterone levels (rho: -0.288, *p* = 0.247). The presence of hypogonadism did not have a significant impact on TMT (hypogonadal patients 6.0 [4.7-7.0] mm, vs. eugonadal patients 6.0 [4.9–6.7] mm; *p* = 0.855). No significant correlation was found between TMT and patients’ weight (rho: 0.228, *p* = 0.064), height (rho: 0.243, *p* = 0.058) or BMI (rho: 0.086, *p* = 0.509).

TMT was higher in patients who had a first MRI closer to the time of diagnosis (i.e. TMT vs. time from diagnosis to first MRI: rho − 0.315, *p* = 0.010).

The presence of hyperprolactinemia, treated hypocortisolism and hypothyroidism, or diabetes mellitus did not significantly affect TMT (all p values > 0.05). Glycated hemoglobin levels showed no statistically significant correlation with TMT (rho: -0.020, *p* = 0.875).

### MMT and patients’ characteristics at first MRI

Patients with active disease at the time of first available MRI had significantly higher MMT values compared to controlled subjects (median 16.2 [14.4–19.0] mm vs. 13.8 [12.4–17.4] mm; *p* = 0.044; Fig. 2E-F), and MMT showed a significant positive correlation with both absolute IGF-1 (rho: 0.393, *p* = 0.002) and IGF-1 xULN values (rho: 0.413, *p* = 0.001). Sex distribution among active disease and controlled patients was almost superimposable (Chi square test, *p* = 0.376). Of note, stratifying patients as of IGF-1 xULN > 2 and IGF-1 ≤ 2 xULN (i.e. clearly uncontrolled disease vs. controlled/mild disease), the difference in MMT values between the two groups was strongly significant (median 17.4 [15.1–19.8] mm vs. 14.7 [12.5–17.3] mm; *p* = 0.005).

MMT was higher in patients who had a first MRI closer to the time of diagnosis (i.e. MMT vs. time from diagnosis to first MRI: rho − 0.368, *p* = 0.004). Furthermore, MMT showed a significant positive correlation with GH values (rho: 0.432, *p* = 0.002). No statistically significant correlation was found between MMT and total testosterone levels (rho: -0.451, *p* = 0.053). The presence of hypogonadism did not significantly affect MMT (hypogonadal patients 15.2 [12.8–17.5] mm vs. eugonadal patients 16.5 [12.5–18.3] mm; *p* = 0.766). MMT showed a positive and significant correlation with both patients’ weight (rho: 0.307, *p* = 0.015) and height (rho: 0.346, *p* = 0.006), while no correlation was found with BMI (rho: 0.200, *p* = 0.139).

Hyperprolactinemia, treated hypocortisolism and hypothyroidism, as well as diabetes mellitus and glycated hemoglobin levels did not have a significant impact on MMT (all p values > 0.05).

### Analysis of all available MRIs

Considering all the MRIs analyzed (*n* = 182), a proper measurement of TMT and MMT was possible in 180 and 158 images, respectively. TMT and MMT values were significantly correlated (rho: 0.526, *p* < 0.001, *n* = 156). This correlation was maintained when analyzing both the subgroup of female (rho: 0.516, *p* < 0.001, *n* = 102) and male patients (rho: 0.345, *p* = 0.011, *n* = 54) (Online Resource 2). At univariable regression analysis, TMT and MMT were positively associated (β 0.540; *p* < 0.001).

Median TMT and MMT values were higher in males compared to females (TMT: 6.5 [5.5–7.5] mm vs. 5.0 [4.5–6.6] mm, *p* < 0.001; MMT: 17.5 [14.5–20.5] mm vs. 14.0 [12.5–16.0] mm, *p* < 0.001). Age at the time of each MRI did not correlate with either TMT or MMT (rho: -0.008, *p* = 0.915 and rho: -0.079, *p* = 0.330; respectively).

TMT showed a significant positive correlation with absolute IGF-1 (rho: 0.182, *p* = 0.015) and IGF-1 xULN values (rho: 0.193, *p* = 0.010), while no significant correlation was found with GH levels (rho: 0.104, *p* = 0.226). No significant correlation was found between TMT and total testosterone values in males (rho: -0.061, *p* = 0.650), and the presence of hypogonadism did not have a significant impact on TMT (hypogonadal patients 5.8 [4.5–6.7] mm vs. eugonadal patients 5.5 [4.7–6.9] mm; *p* = 0.599).

As concerns MMT, we observed a significant positive correlation with absolute IGF-1 (rho: 0.273, *p* = 0.001), IGF-1 xULN values (rho: 0.269, *p* = 0.001), as well as GH levels (rho: 0.213, *p* = 0.019). No significant correlation was found between MMT and total testosterone values in males (rho: -0.190, *p* = 0.195), and the presence of hypogonadism did not have a significant impact on MMT (hypogonadal patients 14.8 [12.7–17.0] mm vs. eugonadal patients 15.0 [12.7–17.9] mm; *p* = 0.519).

### Longitudinal evaluation of TMT and MMT

In patients with at least two MRI evaluations, median time from first to last available MRI was 49 months (IQR 19.0-70.7). Overall, TMT and MMT did not change significantly over time (*p* = 0.157 and *p* = 0.472; respectively). Detailed measures are reported in Online Resource 3.

**Fig. 3:**
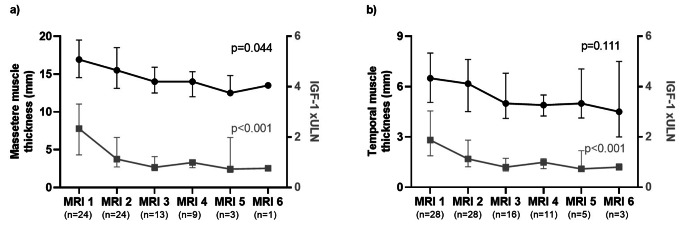
Longitudinal evaluation of MMT and TMT in the patient subgroup with at least two MRI evaluations and active disease (IGF-1 > 1 xULN) at the time of the first MRI (MRI 1) (panels **A-B**). A statistically significant decrease of MMT values was observed during the repeated MRIs, with a consensual significant reduction of IGF-1 xULN over time (panel A). *Abbreviations.* TMT, temporal muscle thickness; MMT, masseter muscle thickness; MRI, magnetic resonance imaging; ULN, upper limit of normality

However, when considering the subgroup of patients with at least two MRI evaluations and active disease (IGF-1 > 1 xULN) at the time of first MRI (MRI 1), we observed a statistically significant decrease of MMT values during the repeated MRIs (MMT baseline 16.9 [14.5–19.5] mm, MMT nadir 12.5 [11.5–14.8] mm; *p* = 0.044, Online Resource 3; Fig. 3A). Of note, we found a consensual significant reduction of IGF-1 xULN levels over time (baseline 2.3 [1.3–3.3] xULN, nadir 0.76 xULN; *p* < 0.001, Fig. 3A). As concerns TMT, a numerical decrease was observed over time (baseline value 6.5 [6.1-8.0] mm, nadir 4.5 [3.0-7.5] mm), but this difference was not statistically relevant; *p* = 0.111 (Online Resource 3, Fig. 4B).

**Fig. 4:**
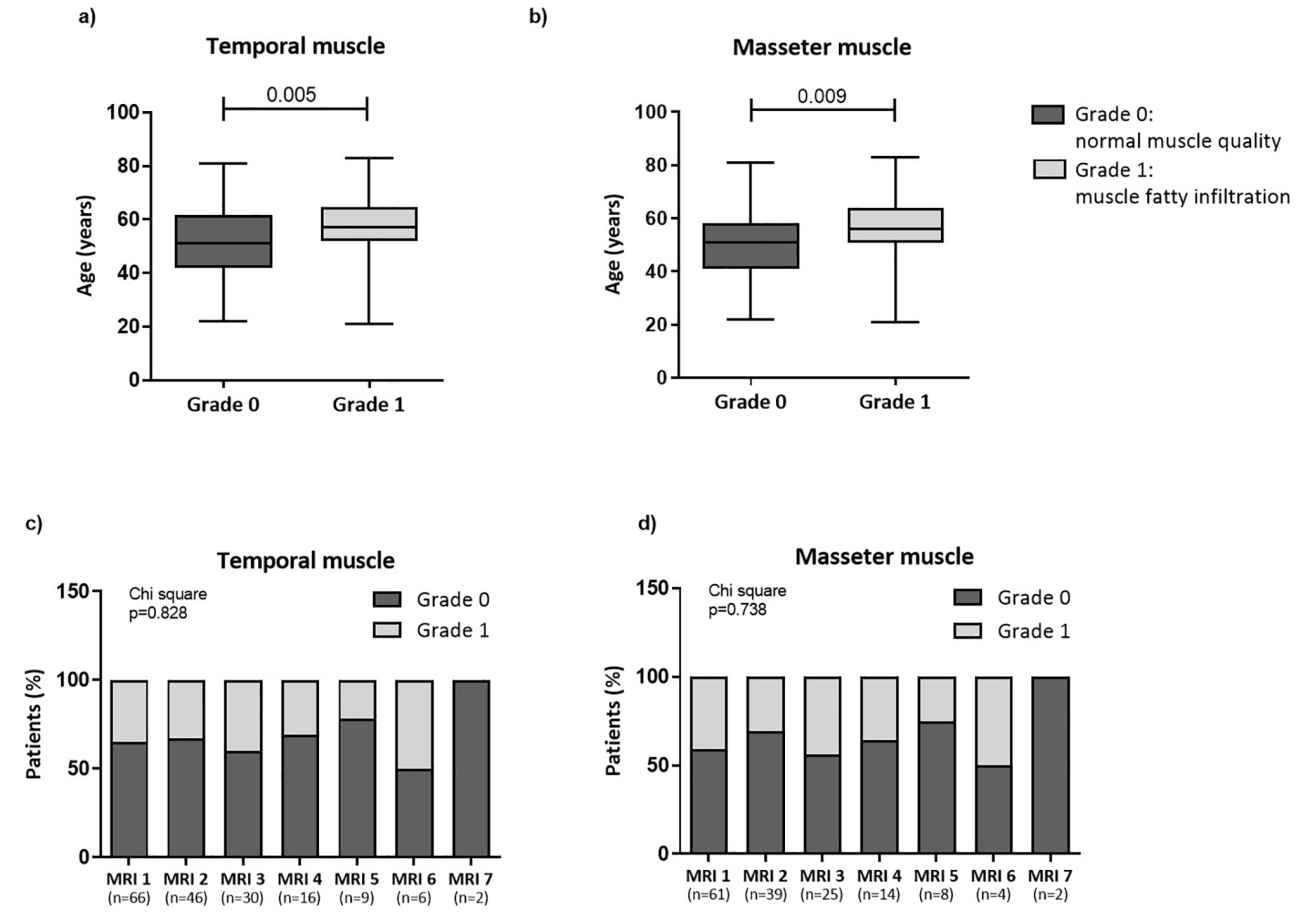
Semi-quantitative evaluation of temporal and masseter muscle fatty infiltration. Patients with a Goutallier grade 1 (remarkable muscle fatty infiltration) were significantly older compared to individuals with normal muscle quality (grade 0). Data depicted in panels **A-B** refer to the evaluation of all available MRIs. The prevalence of MRIs scored as grade 0 or grade 1 did not change significantly during repeated measurements over time for both temporal and masseter muscles (panels **C-D**). *Abbreviations.* TMT, temporal muscle thickness; MMT, masseter muscle thickness; MRI, magnetic resonance imaging

### Determinants of TMT and MMT values

Patients’ sex and IGF-1 xULN values emerged as the main determinants of TMT and MMT. Univariable regression analysis to predict TMT found a negative association with female sex (β -0.349, *p* < 0.001), while a positive association was observed with IGF-1 xULN values (β 0.282, *p* = 0.018) (Online Resource 4). The same finding was observed for MMT, with both female sex (β -0.451, *p* < 0.001) and IGF-1 xULN (β 0.376, *p* < 0.001) identified as significant independent predictors.

The predictive values of these two variables were confirmed at multivariable analysis, showing an adjusted R^2^ of 0.189 for TMT (*p* < 0.001), and 0.312 for MMT (*p* < 0.001, Online Resource 4).

### Temporal and masseter muscle quality

The evaluation of muscle fatty infiltration was performed using a modified Goutallier classification. Considering the first available MRI, 43/66 patients (65%) with a temporal muscle evaluation had grade 0 (normal muscle appearance), while 23 patients (35%) had grade 1 (remarkable fatty infiltration). As concerns masseter muscle, 36/61 patients (59%) had grade 0, while 25 (41%) had grade 1.

When considering all available MRIs, temporal muscle was scored as grade 0 in 115/175 MRIs (66%), while 60 images (34%) had grade 1. Similarly, 96/153 available MRIs (63%) for masseter muscle were scored as grade 0, and 57 images (37%) were scored as grade 1.

The measurement of muscle quality showed a high concordance between temporal and masseter muscles (Κ coefficient: 0.929; *p* < 0.001).

Patients’ age was significantly higher in the MRIs scored as grade 1 compared to those scored as grade 0. This finding was observed for both temporal (57 years [52-64.5] vs. 51 years [42-61.8], *p* = 0.005) and masseter muscles (56 years [50.8–64] vs. 51 years [41–58], *p* = 0.009) (Fig. 4A-B). All the other variables evaluated were not significantly different between MRIs classified as grade 0 and grade 1. At univariable logistic regression analysis, age (stratified into tertiles) was a significant determinant of fatty infiltration for both temporal (OR 1.665, 95% CI 1.112–2.493; *p* = 0.013) and masseter muscle (OR 1.793, 95% CI 1.158–2.777; *p* = 0.009).

Finally, performing the longitudinal evaluation of the different MRIs (from MRI 1 to MRI 7), the prevalence of images scored as grade 0 and grade 1 did not change significantly during repeated measurements over time, for both temporal and masseter muscle (Chi square test: *p* = 0.823 and *p* = 0.712, respectively; Fig. 4C-D).

## Discussion

To our knowledge, this is the first study investigating temporal and masseter muscle thickness, as well as quality, in patients with acromegaly, performing a correlation analysis with clinical, hormonal and demographic characteristics.

TMT is nowadays considered a reliable measure of skeletal muscle mass [17, 18, 27]. This is mainly due to the strong correlation found between TMT and the cross-sectional area (CSA) of the skeletal muscles at the level of the third lumbar vertebra (L3), as well as the psoas muscle CSA, evaluated by use of both CT and MRI scans [16, 28]. Recently, Steindl and colleagues provided age- and sex-related mean TMT reference values studying 624 healthy volunteers [29]. Based on established cut-offs (TMT ≤ 6.3 mm in males and ≤ 5.2 mm in females), about 60% of our patients would have been classified as having normal muscle mass, and 40% as at risk of sarcopenia (data not shown). Therefore, TMT values in patients with acromegaly do not seem to be higher compared to the general population, although a direct comparison was not performed in our study. However, this is in line with the results of a recent systematic review of the literature, showing that in most studies skeletal muscle mass in patients with acromegaly is comparable to that of healthy subjects [15].

MMT has been recently proposed as another independent measure to evaluate skeletal muscle mass, although reference values in the general population are still lacking [30, 31].

Trying to acquire information about patient skeletal muscle mass (and quality) throughout the evaluation of two craniofacial muscles in the context of a pituitary disease, such as acromegaly, provides some clear advantages compared to the investigation of abdominal muscles. All patients with acromegaly perform a brain or sella turcica MRI during their clinical history; first at diagnosis, to identify the presence of a pituitary adenoma, and then during follow-up to monitor the effect of the different treatment approaches [32]. Therefore, our study is based on a “opportunistic” analysis of available images, to retrieve additional information from radiological examinations originally performed for another purpose, with no additional costs and burden for the patients, as well as for the healthcare system.

Temporal muscle can be depicted in its full extent on almost all cranial imaging routinely performed (including brain and sella turcica MRIs), thus limiting the impact of circumstances which could influence muscle thickness (e.g. muscle edema or atrophy) [17, 18, 28]. Since oral and dental diseases could affect TMT and MMT, in line with previous studies the mean of TMT and MMT of both sides (left and right) was used [18]. Craniectomy and radiotherapy can also affect TMT and MMT; however, all the patients included in our cohort had transsphenoidal surgery (if operated), while only one patient underwent conventional radiotherapy.

In line with previous studies, we found that TMT and MMT are strongly and positively correlated each other also in patients with acromegaly [30]. Patients’ sex has been widely demonstrated to impact on TMT, MMT and, more in general, on skeletal muscle mass [30]. Accordingly, in our study we found that male patients had significantly higher TMT and MMT values compared to females. At first MRI, patients’ height and weight were also positively and significantly correlated with MMT. The lack of correlation between TMT, MMT and BMI that we observed in our cohort has been previously reported in other populations, although not unanimously [16, 18, 30, 33].

Temporal muscle is relatively small in its maximum diameter, requiring high accuracy in the measurement and strict adherence to predefined landmarks. Masseter muscle is thicker, and therefore differences between patients (and within the same individual during longitudinal evaluations) can be detected easier. This difference may, at least partially, explain our findings showing stronger correlations/associations between MMT and the different variables evaluated, compared to TMT.

Overall, we found that IGF-1 xULN values were strong predictors of muscle thickness (TMT and MMT), independent of patients’ sex at multivariable regression analysis.

These findings are in line with some previous reports, particularly with the largest study published so far evaluating body composition in 138 patients with acromegaly, by use of DXA [13]. In their manuscript, Reid and colleagues found that both male sex and IGF-1 xULN were positively and significantly associated with the estimated skeletal muscle mass in their cohort, while nor age neither the presence of hypogonadism had a significant impact on it [13]. In line with these findings, in our cohort we observed that TMT and MMT did not significantly correlate with patients’ age nor with the presence of hypogonadism. Although gonadal steroids are important modulators of body composition, different authors did not find a significant association between changes in this parameter and skeletal muscle mass in patients with acromegaly [13, 34]. This could be due to the strong impact of GH/IGF-1 axis on gonadal function, and the significant inverse correlation observed between IGF-1 and total testosterone levels in males, that we also found in our cohort.

The association between IGF-1 xULN and skeletal muscle mass is strengthened by the longitudinal evaluation of our patients, since the decrease in IGF-1 levels observed during follow-up was mirrored by a related decrease in MMT (regardless of the patients’ age at the time of subsequent MRI scans).

Since it has been widely-demonstrated that patients with acromegaly have impaired muscle quality, we performed a semi-quantitative evaluation of fatty infiltration on temporal and masseter muscles. We found that 35–40% of our patients have remarkable fatty infiltration of both muscles evaluated. Previous studies have investigated the presence and the amount of fatty infiltration in patients with acromegaly by use of various techniques, providing measures of different parameters, such as the intermuscular adipose tissue (IMAT) [4, 10, 14, 34, 35]. Most studies report an increased IMAT in patients with acromegaly compared to controls, with the highest values observed in untreated males [4, 10]. Overall, surgical or medical treatment does not result in a significant lowering of IMAT values, despite a marked decrease in GH and IGF-1 levels [10, 14]. In our cohort we observed that age was the main determinant of muscle fat infiltration, with older patients being more likely to have remarkable fat infiltration. This is in line with a number of previous studies demonstrating an increasing muscle fat content with age in non-acromegaly patients [36, 37]. In this light, our data suggest that, in patients with acromegaly, age could have a bigger impact on muscle quality than on muscle size or mass [36].

We acknowledge that our study has some limitations, mainly due to the retrospective study design and the lack of a control group of healthy subjects. Muscle fatty infiltration has been measured by a semi-quantitative method, which provides limited information compared to IMAT evaluation (although this latter requires a specific evaluation and additional resources).

The longitudinal evaluation performed in most patients gave us additional information and strengthened our findings. Considering the global prevalence of acromegaly, we believe that the number of patients included, as well as the collection of about 180 MRI examinations, are adequate to address the aims of this study.

In conclusion, based on the recent assumption that both TMT and MMT are reliable measures of skeletal muscle mass, we demonstrated that sex and IGF-1 xULN values are the main determinants of muscle thickness in patients with acromegaly. Male patients with higher IGF-1 values have thicker temporal and masseter muscles, and, therefore, they have more skeletal muscle mass compared to the other patients. Patients’ age has a greater impact on muscle quality than on muscle mass, while the presence of hypogonadism or impaired glucose metabolism do not significantly affect both evaluations. Further studies are needed to better investigate whether TMT and MMT, assessed from a brain/sella turcica MRI, can provide additional information, showing some degree of association with patients’ quality of life, disease-related symptoms, as well as the presence and the severity of various comorbidities linked to acromegaly.

## Electronic supplementary material

Below is the link to the electronic supplementary material.

Supplementary Material 1

Supplementary Material 2

Supplementary Material 3

Supplementary Material 4

## Data Availability

The datasets generated during and analyzed during the current study are not publicly available due to reason of sensitivity but are available from the corresponding author on reasonable request.
