# Supplementary material for: Temporal and masseter muscle evaluation by MRI provides information on muscle mass and quality in acromegaly patients
Source: Pituitary. 2024 Jul 5;27(5):507–17. doi: 10.1007/s11102-024-01422-y (PMC11513697; doi:10.1007/s11102-024-01422-y)
Supplement: Supplementary file 1 — Supplementary Material 1 [file 11102_2024_1422_MOESM1_ESM.pdf]

Pituitary Journal

Temporal and masseter muscle evaluation by MRI provides information on muscle mass and quality in acromegaly patients

Federico Gatto[1], Angelo Milioto[1,2], Giuliana Corica[1,2], Federica Nista[3], Claudia Campana[2], Anna Arecco[2], Lorenzo Mattioli[2], Lorenzo Belluscio[2], Bianca Bignotti[3], Diego Ferone[1,2], Alberto Stefano Tagliafico[3,4]

[1]Endocrinology Unit, IRCCS Ospedale Policlinico San Martino, Genoa, Italy

[2]Endocrinology Unit, Department of Internal Medicine and Medical Specialties (DIMI) and Centre of Excellence for Biomedical Research (CEBR), University of Genova, Genoa, Italy

[3]Radiology Section, Department of Health Sciences (DISSAL), University of Genova, Genoa, Italy

[4]Department of Radiology, IRCCS Ospedale Policlinico San Martino, Genoa, Italy

Corresponding author:

Federico Gatto, MD, PhD

Email fedgatto@hotmail.it; federico.gatto@hsanmartino.it

Supplemental patient information

- **Glucose metabolism**

The presence of diabetes mellitus was reported in 14 out of 69 patients (20.3%), with 10/14 individuals undergoing antidiabetic treatment (i.e. metformin monotherapy, n=5; metformin plus sulfonylurea, n=1; metformin plus SGLT-2 inhibitor, n=1; metformin plus DPP-4 inhibitor, n=1; metformin plus SGLT2 inhibitor (daily) plus GLP-1 receptor agonist (weekly), n=1; insulin, n=1).

- **Clinical history of cancer**

Thirteen patients had a clinical history of cancer (13/69, 18.8%; prostate cancer, n=3; papillary thyroid carcinoma, n=2; parotid gland adenocarcinoma, n=1; rectum adenocarcinoma, n=1; renal cancer and nasopharynx cancer, n=1; basocellular skin carcinoma, n=1; melanoma, n=1; lung cancer, n=1; endometrial cancer, n=1; gastric cancer, n=1).
