# Supplementary material for: Temporal and masseter muscle evaluation by MRI provides information on muscle mass and quality in acromegaly patients
Source: Pituitary. 2024 Jul 5;27(5):507–17. doi: 10.1007/s11102-024-01422-y (PMC11513697; doi:10.1007/s11102-024-01422-y)
Supplement: Supplementary file 2 — Supplementary Material 2 [file 11102_2024_1422_MOESM2_ESM.pdf]

Pituitary Journal

Temporal and masseter muscle evaluation by MRI provides information on muscle mass and quality in acromegaly patients

Federico Gatto[1], Angelo Milioto[1,2], Giuliana Corica[1,2], Federica Nista[3], Claudia Campana[2], Anna Arecco[2], Lorenzo Mattioli[2], Lorenzo Belluscio[2], Bianca Bignotti[3], Diego Ferone[1,2], Alberto Stefano Tagliafico[3,4]

[1]Endocrinology Unit, IRCCS Ospedale Policlinico San Martino, Genoa, Italy

[2]Endocrinology Unit, Department of Internal Medicine and Medical Specialties (DIMI) and Centre of Excellence for Biomedical Research (CEBR), University of Genova, Genoa, Italy

[3]Radiology Section, Department of Health Sciences (DISSAL), University of Genova, Genoa, Italy

[4]Department of Radiology, IRCCS Ospedale Policlinico San Martino, Genoa, Italy

Corresponding author:

Federico Gatto, MD, PhD

Email fedgatto@hotmail.it; federico.gatto@hsanmartino.it

Supplementary Figure 1. Correlation between TMT and MMT, considering all evaluated MRIs

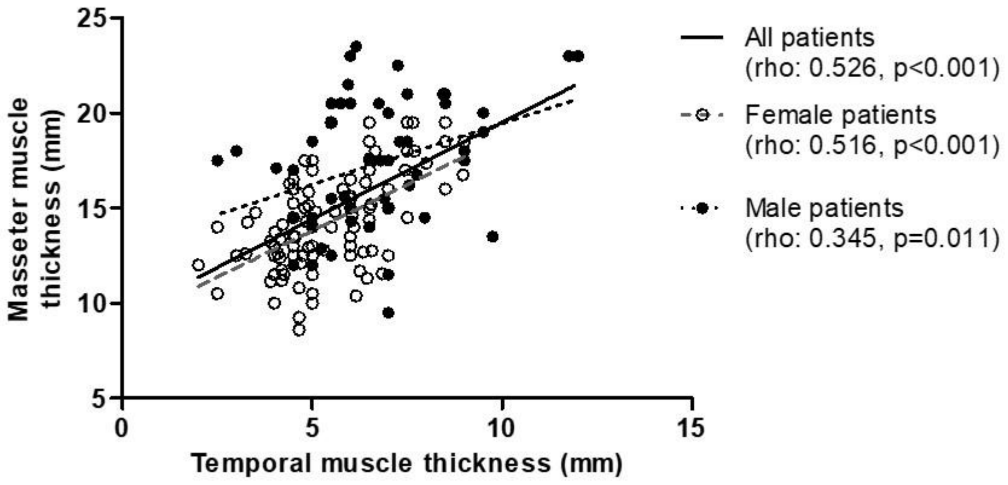

Supplementary Figure 1. Correlation between TMT and MMT, considering all available MRIs. The correlation was maintained when analyzing both the subgroups of female and male patients. *Abbreviations.* TMT, temporal muscle thickness; MMT, masseter muscle thickness, MRI, magnetic resonance imaging.
