# Supplementary material for: Temporal and masseter muscle evaluation by MRI provides information on muscle mass and quality in acromegaly patients
Source: Pituitary. 2024 Jul 5;27(5):507–17. doi: 10.1007/s11102-024-01422-y (PMC11513697; doi:10.1007/s11102-024-01422-y)
Supplement: Supplementary file 3 — Supplementary Material 3 [file 11102_2024_1422_MOESM3_ESM.docx]

**Pituitary Journal**

**Temporal and masseter muscle evaluation by MRI provides information on muscle mass and quality in acromegaly patients**

Federico Gatto^1^, Angelo Milioto^1,2^, Giuliana Corica^1,2^, Federica Nista^3^, Claudia Campana^2^, Anna Arecco^2^, Lorenzo Mattioli^2^, Lorenzo Belluscio^2^, Bianca Bignotti^3^, Diego Ferone^1,2^, Alberto Stefano Tagliafico^3,4^

^1^Endocrinology Unit, IRCCS Ospedale Policlinico San Martino, Genoa, Italy

^2^Endocrinology Unit, Department of Internal Medicine and Medical Specialties (DIMI) and Centre of Excellence for Biomedical Research (CEBR), University of Genova, Genoa, Italy

^3^Radiology Section, Department of Health Sciences (DISSAL), University of Genova, Genoa, Italy

^4^Department of Radiology, IRCCS Ospedale Policlinico San Martino, Genoa, Italy

**Supplementary Table 1.** Longitudinal evaluation of TMT and MMT over time, as well as IGF-1 xULN values, in patients with at least
two MRI evaluations.

| **Muscle measurement** | **MRI evaluation** | | | | | | |  |
| --- | --- | --- | --- | --- | --- | --- | --- | --- |
| **All patients** | **MRI 1** | **MRI 2** | **MRI 3** | **MRI 4** | **MRI 5** | **MRI 6** | **MRI 7** | **p value** |
| **TMT**  median (IQR); mm | 6.1  (4.9-7.5) | 5.9  (4.6-7.5) | 5.3  (4.2-7.0) | 4.9  (4.4-5.6) | 5.0  (4.4-5.8) | 4.75  (3.7-6.0) | 6.3  (6.0-6.5) | 0.157 |
| **IGF-1xULN**  median (IQR) | 1.11  (0.80-2.45) | 0.88  (0.63-1.29) | 0.70  (0.48-0.96) | 0.83  (0.64-1.1) | 0.70  (0.63-0.89) | 0.78  (0.48-0.94) | 0.62  (0.53-0.70) | **<0.001** |
| Pt. number^a^ | n=47 | n=46^b^ | n=29 | n=17 | n=13 | n=6 | n=2 |  |
| **MMT**  median (IQR); mm | 16.0  (13.0-18.5) | 15.0  (12.9-19.3) | 14.0  (12.6-16.7) | 14.2  (12.6-17.0) | 13.4  (12.5-15.0) | 14.0  (11.7-15.3) | 16.3  (15.0-17.5) | 0.472 |
| **IGF-1xULN**  median (IQR) | 1.18  (0.78-3.05) | 0.88  (0.62-1.47) | 0.70  (0.51-0.93) | 0.83  (0.65-1.14) | 0.66  (0.55-1.04) | 0.62  (0.45-0.90) | 0.62  (0.53-0.70) | **0.003** |
| Pt. number^a^ | n=42 | n=37^c^ | n=24 | n=15 | n=7 | n=4 | n=2 |  |
| **Active disease**  **at MRI 1** | **MRI 1** | **MRI 2** | **MRI 3** | **MRI 4** | **MRI 5** | **MRI 6** | **MRI 7** | **p value** |
| **TMT**  median (IQR); mm | 6.5  (5.1-8.0) | 6.2  (3.5-4.5) | 5.0  (2.5-4.1) | 4.3  (4.1-4.9) | 4.1  (3.5-5.0) | 3.0  (3.0-4.5) | n.a | 0.111 |
| **IGF-1xULN**  median (IQR) | 1.88  (1.25-3.03) | 1.13  (0.82-1.88) | 0.80  (0.65-1.15) | 0.99  (0.74-1.14) | 0.73  (0.65-1.15) | 0.80  (0.76-0.93) | n.a | **<0.001** |
| Pt. number^a^ | n=28 | n=28 | n=16 | n=11 | n=5 | n=3 |  |  |
| **MMT**  median (IQR); mm | 16.9  (14.5-19.5) | 15.5  (13.1-18.5) | 14.0  (12.5-15.9) | 14.0  (12.0-15.3) | 12.5  (11.5-14.8) | 13.5 | n.a. | **0.044** |
| **IGF-1xULN**  median (IQR) | 2.34  (1.29-3.31) | 1.13  (0.82-1.99) | 0.80  (0.67-1.23) | 0.99  (0.78-1.10) | 0.73  (0.60-1.98) | 0.76 | n.a | **<0.001** |
| Pt. number^a^ | n=24 | n=24 | n=13 | n=9 | n=3 | n=1 |  |  |

**Supplementary Table 1.** Longitudinal evaluation of TMT and MMT over time, as well as IGF-1 xULN values, in patients with at least
two MRI evaluations.

*Abbreviations.* TMT, temporal muscle thickness; MMT, masseter muscle thickness; ULN, upper limit of normality; MRI, magnetic resonance imaging; Pt., patient; IQR, interquartile range; mm, millimeter; n.a., not available.

^a^As mentioned in the text, a proper measurement of both TMT and MMT, according to the described Methods, was not possible in all MRIs. ^b^One patient had TMT measurement available at MRI 1 and MRI 4. ^c^Five patients had MMT measurements available at MRI 1 and the following ones at MRI 3 (four cases) and MRI 4 (one case).
