# Supplementary material for: Temporal and masseter muscle evaluation by MRI provides information on muscle mass and quality in acromegaly patients
Source: Pituitary. 2024 Jul 5;27(5):507–17. doi: 10.1007/s11102-024-01422-y (PMC11513697; doi:10.1007/s11102-024-01422-y)
Supplement: Supplementary file 4 — Supplementary Material 4 [file 11102_2024_1422_MOESM4_ESM.pdf]

Pituitary Journal

Temporal and masseter muscle evaluation by MRI provides information on muscle mass and quality in acromegaly patients

Federico Gatto[1], Angelo Milioto[1,2], Giuliana Corica[1,2], Federica Nista[3], Claudia Campana[2], Anna Arecco[2], Lorenzo Mattioli[2], Lorenzo Belluscio[2], Bianca Bignotti[3], Diego Ferone[1,2], Alberto Stefano Tagliafico[3,4]

[1]Endocrinology Unit, IRCCS Ospedale Policlinico San Martino, Genoa, Italy

[2]Endocrinology Unit, Department of Internal Medicine and Medical Specialties (DIMI) and Centre of Excellence for Biomedical Research (CEBR), University of Genova, Genoa, Italy

[3]Radiology Section, Department of Health Sciences (DISSAL), University of Genova, Genoa, Italy

[4]Department of Radiology, IRCCS Ospedale Policlinico San Martino, Genoa, Italy

Corresponding author:

Federico Gatto, MD, PhD

Email fedgatto@hotmail.it; federico.gatto@hsanmartino.it

Supplementary Table 2. Univariable and multivariable linear regression analyses for the prediction of TMT and MMT (all available MRIs).

**Univariable linear regression analyses**						
Dependent Variables	Independent Variables (IVs)	Adjusted R^2	B	B 95% CI	β	p value
**TMT** (mm)	Sex, F	0.117	-1.235	-1.726, -0.744	-0.349	**<0.001**
	IGF-1 xULN	0.074	0.579	0.284, 0.874	0.282	**<0.001**
**MMT** (mm)	Sex, F	0.196	-2.990	-3.926, -2.053	-0.451	**<0.001**
	IGF-1 xULN	0.136	1.295	0.783, 1.806	0.376	**<0.001**
**Multivariable linear regression analyses**						
Dependent Variables	Independent Variables (IVs)	Adjusted R^2	B	B 95% CI	β	p value
**TMT** (mm)	All IVs	0.189	-		-	**<0.001**
	Sex, F	-	-1.232	-1.712, -0.751	-0.345	**<0.001**
	IGF-1 xULN	-	0.528	0.251, 0.805	0.257	**<0.001**
**MMT** (mm)	All IVs	0.312	-		-	**<0.001**
	Sex, F	-	-2.836	-3.724, -1.949	-0.426	**<0.001**
	IGF-1 xULN	-	1.129	0.670, 1.588	0.328	**<0.001**

Supplementary Table 2. Univariable and multivariable linear regression analyses for the prediction of TMT and MMT (all available MRIs).
Abbreviations. TMT, temporal muscle thickness; MMT, masseter muscle thickness; mm, millimeter.
